# Stem Cells in Equine Veterinary Practice—Current Trends, Risks, and Perspectives

**DOI:** 10.3390/jcm8050675

**Published:** 2019-05-14

**Authors:** Katarzyna Kornicka, Florian Geburek, Michael Röcken, Krzysztof Marycz

**Affiliations:** 1 Department of Experimental Biology, The Faculty of Biology and Animal Science, University of Environmental and Life Sciences, 50-375 Wroclaw, Poland; kornicka.katarzyna@gmail.com; 2 International Institute of Translational Medicine, Malin, Jesionowa 11, 55-114 Wisznia Mała, Poland; 3 Faculty of Veterinary Medicine, Equine Clinic-Equine Surgery, Justus-Liebig-University, 35392 Giessen, Germany; florian.geburek@vetmed.uni-giessen.de (F.G.); michael.roecken@vetmed.uni-giessen.de (M.R.)

**Keywords:** adipose derived stem cells, extracellular microvesicles, regenerative medicine

## Abstract

With this Editorial, we introduce the Special Issue “Adipose-Derived Stem Cells and Their Extracellular Microvesicles (ExMVs) for Tissue Engineering and Regenerative Medicine Applications” to the scientific community. In this issue, we focus on regenerative medicine, stem cells, and their clinical application.

Recently, stem cell-based therapies have been widely applied in the equine veterinary field [1]. For more than a decade, the transplantation of autologous mesenchymal stem cells (MSCs) has been investigated in multiple experimental and clinical animal trials worldwide [2]. MSCs have been shown to have positive effects in the treatment of musculoskeletal, neurodegenerative, metabolic, and immune diseases [3]. The clinical application of MSCs has raised hope for a more effective treatment of tendon and articular diseases, which are the most common musculoskeletal disorders in horses [4]. The unique properties of MSCs, i.e., multipotency, proliferative, and clonogenic potential and paracrine action, make them an innovative tool for improved repair or even potential regeneration of damaged tissue [3]. During the last 20 years, several research groups have investigated various aspects of MSC biology in the context of their clinical usefulness. MSCs can be isolated from multiple tissue sources, but most attention has been paid to cells isolated from bone marrow (BM-MSCs), adipose tissue (AT-MSCs/ASCs), Wharton’s jelly (WJMSCs) [5], and, more recently, blood [6]. The source selection of the stem cells depends on both ease of access and of harvesting, the need for local or general anesthesia, and, finally, yield and quality of the isolated cells. Further research by molecular biologists has focused on cytophysiological aspects of MSCs: their metabolic activity, presence of particular surface antigens, gene expression profile, and proteomics. Adult MSCs from diverse sources behave in predictable ways, which offer promise in terms of their clinical applications [7]. Early publications showed the beneficial effects of autologous ASCs and BMSCs transplantation on tendinopathies of the equine superficial digital flexor tendon [8,9]. For example, it was shown that autologous ASCs enhance perfusion and neovascularization of healing experimental tendon lesions in horses [10]. Recently, other studies have delivered clinical evidence that autologous MSCs applied by intralesional injection, intravenously, and, more recently, intraarterially, can be an effective therapeutic approach in the treatment of tendinopathies when compared with conventional treatments, e.g., anti-inflammatory drugs [11,12,13,14]. However, the effect of a single intralesional treatment with autologous MSCs has proved to be limited in an equine experimental model of tendinopathy [15]. Several studies have investigated the effects of MSCs combined with platelet rich plasma (PRP) or other blood-based substrates to improve the clinical outcome and prolong therapeutic effects [16,17]. After years of MSC research, there remain discrepancies between promising experimental in vitro and in vivo results and evidence-based safety as well as clinical effectivity of such therapies. Multiple factors affect MSCs properties, including their niche, which may reduce their therapeutic potential [18,19,20,21]. Current molecular studies have revealed that the regenerative potential of MSCs strongly depends on the age and metabolic condition of a patient, including insulin resistance [22,23]. Time and temperature of MSC shipment as well as application technique are crucial for its viability [24]. However, specific data regarding these factors is lacking in the literature. The development of a “passport” for stem cells that describes their physiological condition prior to their clinical application is crucial to standardize the procedure. This document should include information regarding the source of the MSCs as well as phenotype, proliferative potential, apoptotic genes expression, clonogenic potential, multipotency, as well as the time and temperature of shipment and storage before clinical application of the cells. The description of these factors should be a minimum requirement before cell transplantation. 

Autologous MSCs transplantation will never be a true “off the shelf” therapy because it requires time for cell isolation, additional laboratory work, and, most importantly, a minimum of 10 days of propagation in vitro to obtain the number of MSCs estimated to be adequate for clinical application [25]. MSCs were previously thought to be immune privileged and considerable attention has been paid towards allogenic therapies in equids and other mammals [26,27]. The perspective of immediate application of MSCs was attractive to the veterinary community because it allowed for patient treatment without loss of time or risk of disease progression. Lack of MHC II in MSCs was proved in multiple studies; thus, MSCs are considered safe in cases of allogeneic administration [28]. However, there have been several reports of adverse clinical events in equine models [29,30]. Modification of culture condition, a 48-hour depletion culture period of fetal bovine serum (FBS), greatly eliminates the risk of adverse effects [30]. Another option may be ex vivo adaptation of MSCs in autologous serum-supplemented medium prior to application [31]. The strategy to apply allogeneic MSCs is attractive from a clinical perspective; however, a proper cell culture method is required to eliminate potential risk. 

Stem cells therapies in equine veterinary practice have been mostly applied for the treatment of disorders of the musculoskeletal system [2,32]. However, recent data has shown that MSCs are potentially effective in the treatment of other diseases in equids including insulin resistance (IR) [33,34]. Several studies have shown that both obesity and IR negatively affect multipotency of MSCs through impairment of autophagy, a process which allows stem cells to remove dysfunctional organelles and regenerate [35,36,37,38]. It has been shown that ASCs derived from IR horses are characterized by elevated oxidative stress and aged phenotype which may disqualify them for clinical application [37]. Therefore, it is necessary to rejuvenate impaired autologous MSCs prior to clinical application. Several chemicals with antioxidative and anti-aging properties have been proposed as rejuvenating agents; these include 5-azacytidine (demethylation agent) and resveratrol (polyphenol). It has been shown that a combination of these substances successfully reverses the aged nature of ASCs derived from IR horses [39,40,41]. This phenomenon may contribute to the development of a new branch in veterinary pharmacotherapy, i.e., stem cell pharmacology. In the near future, pre-treatment of MSCs with pharmacological substances will likely become a common procedure for the modulation of cytophysiological properties of stem cells before their clinical application. 

Initially, the hypothesis behind MSC therapy suggested that viable cells integrate in the tissue defect to replace it. Although it could be shown experimentally that high numbers of MSCs remained in and near equine experimental tendon lesions after local application [42], the percentage of cells injected significantly decreased shortly after application depending on the cell type [43]. Accordingly, the secretome of MSCs and the mechanisms by which it affects damaged tissues has been under intense investigation recently [44,45]. Several research groups showed that MSCs secrete a wide range of growth factors, chemokines, and cytokines, which are either released into intercellular space or transported in extracellular membrane-derived microvesicles (ExMVs) to neighboring cells [46]. Recently, it was shown that ExMVs cargo also contains miRNAs, which indirectly or directly modulate gene expression in recipient cells of damaged tissue [47]. Evidence suggests that the secretomes of MSCs constantly promote regeneration of damaged tissue by various mechanisms, including inhibition of apoptosis, promotion of cell survival, and, most importantly from a clinical perspective, exertion of immunomodulatory effects [48]. Moreover, the secretomes of MSCs promote neurogenesis and angiogenesis, which may be fundamental in the course of the regenerative process because “there is no regeneration without vascularization” [49]. Thus, future research in equine regenerative medicine should focus on the regeneration of damaged tissue by the application of MSCs secretomes or their elements because, unlike allogenic MSCs, they are not expected to cause any side effects. On the basis of recent findings, one might speculate that the application of MSCs secretomes might become a useful therapeutic tool in equine regenerative medicine in the near future. 

Stem cells have brought new hope for veterinary regenerative medicine and are becoming an increasingly promising clinical tool. However, many questions remain unanswered, including the justifiability of allogenic stem cell application or the clinical utility of MSCs isolated from individuals diagnosed with certain disorders. There are strong requirements justifiable for further consolidation grants to elaborate exact protocols for the molecular and physiological characteristics of MSCs prior to MSCs-based experimental and clinical animal trials as well as, ultimately, routine clinical application.
